# The impact of a dedicated training program for oral examiners at a medical school in Germany: a survey among participants from operative and non-operative disciplines

**DOI:** 10.1186/1754-9493-7-22

**Published:** 2013-07-03

**Authors:** Wolfgang Oechsner, Sandra Geiler, Markus Huber-Lang

**Affiliations:** 1 Department for Cardiac Anaesthesiology, University Hospital Ulm, Ulm, Germany; 2 Department for Evaluation and Quality Management in Medical Education, Medical Faculty of the University of Ulm, Ulm, Germany; 3 Clinic for Trauma Surgery, Hand Surgery, Plastic Surgery and Reconstructive Surgery, University Hospital Ulm, Ulm, Germany

**Keywords:** Oral examinations, Patient safety, Examiner trainings, Medical education

## Abstract

**Background:**

Oral examinations have been a crucial format in ancient and modern assessment to evaluate and guarantee quality of medical education and thereby to secure patient safety. To achieve a high level of quality in the oral part of the final examination of medical students, a training program for oral examiners at the Medical Faculty of Ulm (Germany) has been established since 2007.

However, little is known about the attitude of the examiners in regard to the impact of this training program and of oral examinations as instruments to ensure patient safety.

**Methods:**

All 367 academic clinicians from operative and non-operative disciplines, attending the one-day examiner training program at the University of Ulm between 2007 and 2012 have been asked to answer an online survey (EvaSys 5.0). Focus of the survey was to find out in which respect the examiners profited from the trainings, if the training effects were discipline-dependent, and to which degree the oral examinations could contribute to patient safety. Statistical analysis was performed using the t-test for independent samples. Results were considered statistically significant when p < 0.05.

**Results:**

A total of 63 participants answered the survey, but in 4 cases the questionnaire was not fully completed (with single items missing). More than half of the study participants (n = 34/59; 58%) have experienced (at least sometimes or rarely) candidates that they deemed incompetent and perhaps even dangerous to the patients’ health who nevertheless passed the oral exam successfully. The majority of participants were convinced that oral examinations using concrete clinical cases could significantly contribute to patient safety, if grading is based on clear criteria and if examinations as well as grading are performed more critically. The impact of the training program was rated significantly stronger by surgeons than by non-surgeons in several categories. These categories included “strengths and weaknesses of oral examinations”, “reliability”, “validity”, “competence in grading”, “critical grading”, and “departmental improvements” concerning oral examinations.

**Conclusions:**

In respect to patient safety, it seems crucial to prevent incompetent candidates from passing the oral examination. The present study indicates the importance to continue and to develop our examiner trainings, with main emphasis on concrete clinical problems and a criteria-based critical grading system for oral examinations. Since the impact of the training was particularly high for colleagues from the operative disciplines, the training program should be offered especially in surgical departments.

## Background and study goals

Besides various oral and written feedback mechanisms during medical school, the final examination of medical students represents a medical and legal tool to assure a certain level of quality for future patient care. In this context medical students in Germany have to pass two parts in their final exams: part 1 contains a series of multiple choice questions, part 2 consists of an oral examination performed and graded by medical experts from operative and non-operative disciplines [1].

In the written part, all questions are identical for all candidates throughout the country, covering all important medical topics. Those multiple-choice questions are known to examine the students’ medical knowledge with a high level of reliability [2].

In the oral part of the examination, each examiner is free to challenge the student with individual medical tasks (e.g. questions concerning medical problems, clinical reasoning, systematic medical knowledge, or practical procedures). However, neither the content, nor the structure, nor the expected level of knowledge/expertise, nor the criteria for grading are pre-defined. This lack of central structuring and regulation is somewhat surprising, as the grade for the oral part of the exam contributes significantly to the overall grade in the German final medical exam. High reliability and high validity are demanded as the most important criteria for “high stakes” examinations such as the final exam in medicine. In contrast, low reliability and low validity are described as consequences of poorly structured oral examinations [3-6].

To improve these potential weaknesses of oral medical examinations, the Medical Faculty of the University of Ulm has been offering (since 2007) special trainings for examiners involved in the oral part of the final exam. The main goal of these trainings is to enable the examiners to master the technique of the structured oral examination (i.e. construction of clinically relevant cases, tasks and problems as well as defining the respective level of expectation and its communication to co-examiners). Furthermore, the examiners should acquire knowledge about factors that influence reliability and validity of oral examinations. As a proof of concept, a recent evaluation of this examiner training program (in which all participants between 2007 and 2012 were addressed to answer an online survey regarding the effects of the trainings and the sustainability of the training effects) revealed six main effects in the following categories [7]:

1. Conscious handling of both the strengths and weaknesses of oral examinations

2. Knowledge of the factors influencing the reliability of oral exams

3. Knowledge of the factors influencing the validity of oral exams

4. Competence in the construction of oral examination tasks

5. Knowledge of the formal and legal regulations of the oral part of the final exam

6. Implementing the concept of structured oral examinations in the final exam

These findings are supported by others, indicating that the quality of oral examinations can be improved by specific educational measures [4,8,9].

However, little is known about differences in the benefit of examiner trainings in relation to the examiners’ professional background, and about their experiences and attitudes in respect to the students’ examination performance and probable consequences for patient safety.

Therefore, the present study was designed to find out to which degree (according to the examining colleagues) the oral examination component of the final medical school exam may contribute to patient safety, and if the answers of the colleagues from operative disciplines differ from the answers of those from non-operative disciplines in respect to the effect of the offered trainings.

## Methods

All examiner trainings at the University of Ulm have been conducted since 2007 until present as a one day workshop led by the same certified trainer. The trainer is a member of faculty in the Department of Cardiac Anaesthesiology and holds an additional master degree in medical education (MME Bern, Switzerland). The trainees are clinical experts from non-operative and operative disciplines, belonging to the University Hospital Ulm or associated academic hospitals. The participation was voluntary. The training consisted of a seminar with oral presentations, discussions, individual and group work, and an examination simulation with feedback for the participants from peers and experts.

All participants (n = 367) in these trainings from 2007 to 2012 were contacted online and asked to complete a quantitative survey which was performed with the help of the tool EvaSys 5.0. Because of the general fluctuation at any University Hospital, many of the former trainees may not have received the online request. A total of 63 attendees of the examiner training answered the present survey and were defined as the study participants. As indicated in the results part, minimal variations in the total n-size were caused by questionnaires not fully completed. All data were registered and handled anonymously.

The survey consisted of a total of 28 items. One focus covered items concerning effectiveness and sustainability of the training and has been recently accepted for publication [7]. The focus of present study was on items that referred to the contribution of oral examinations and of the examiner trainings in regard to patient safety and on specific demographic data of the professional background.

Likert scales were used when applicable. The data are presented as mean +/- SD. Statistical analysis was performed using the t-test for independent samples. Results were considered statistically significant when p < 0.05.

### Consent and ethical approval

According to our university guidelines and to the local Independent Ethical Committee of the University of Ulm no specific ethical approval was required to perform the study. Therefore, a written consent was not necessary. However, all study steps were performed strictly in accordance with the Helsinki Declaration.

## Results

### Oral examiners experienced risky incompetence of the candidates

More than half of the study participants (n = 34/59; 58%) have experienced incompetent candidates who have nevertheless passed the exam successfully. More specifically, the following survey item: “I have experienced that candidates who, in my opinion, have been incompetent and perhaps even dangerous to the patients’ health yet passed the exam” was answered by 20% (n = 12/59) of the examiners with “sometimes” and by 37% (n = 22/59) with “rarely”. Only 42% (n = 25/59) responded to have “never” experienced such a situation in their final exams. None of the participants answered with the option “frequently”.

### Proposed link between performance of oral examinations and patient safety

All items concerning the topic “patient safety” could be answered by the participants on a 6-step Likert scale with 1 = applicable until 6 = not applicable. Table 1 shows the number of participants clearly affirming the respective item by choosing 1 or 2 on this scale. The number of participants choosing step 3 is also displayed as “tendency towards confirmation”.

**Table 1:** Items in respect to patient safety (n = 62-63 answers per item)

**Item**	**Number of answers with clear affirmation (scale 1–2)**	**Number of answers with tendency towards affirmation (Scale 3)**	**Cumulated answers (Scale 1–3)**
Oral examinations using concrete clinical cases or problems essentially contribute to patient safety.	42/63 (67%)	9/63 (14%)	51/63 (81%)
For patients’ safety it is important to have clear and criteria-based rules for grading in the oral part of the final exam.	34/63 (54%)	14/63 (22%)	48/63 (76%)
For patients’ safety the candidates should be examined more critically in the oral part of the final exam.	39/62 (63%)	11/62 (18%)	50/62 (81%)

The majority of participants (67%; n = 42/63) seemed to be clearly convinced that oral examinations using concrete clinical cases significantly contribute to patient safety. Furthermore, more than half of the participants (54%; n = 34/63) definitely agreed that for this aim it is important to have clear and criteria-based rules for grading. Finally, most examiners (63%; n = 39/62) strongly agreed that for patient safety issues the candidates should be examined more critically (*see* Table 1).

### Differences between operative and non-operative disciplines in evaluation of examiner trainings

Using the demographic data of the survey, the effects of the examiner trainings were analysed in respect to the participants’ professional background. The answers were obtained on a 6 step Likert scale (1 = applicable, 6 = not applicable) and analysed for differences between the subgroups of participants from operative (44%; n = 28/63) versus non-operative (56%; n = 35/63) disciplines.

In regard to the main effects of the examiner trainings (described in the introduction part) significant differences were found in the following three topics: conscious handling of the strengths and the weaknesses of oral examinations, knowledge of the factors that influence the reliability of oral examinations, knowledge of the factors that influence the validity of oral examinations (*see* Table 2).

**Table 2:** Differences between operative and non-operative disciplines in evaluation of examiner trainings, concerning the main effects of the training

**Professional discipline**		**N**	**Mean**	**SD**	**p-value**	**T**	**df**	**Mean difference**
The training contributed to conscious handling of both the strengths and weaknesses of oral examinations	Operative	28	1.9	1.1				
	Non-operative	35	2.6	1.5	0.04	-2.1	61	-0.7
The training led to profound knowledge of the factors influencing the reliability of oral exams	Operative	28	1.9	0.9				
	Non-operative	34	2.7	1.5	0.01	-2.7	57	-0.8
The training led to profound knowledge of the factors influencing the validity of oral exams	Operativ	28	1.9	1.0				
	Non-operative	35	2.6	1.4	0.03	-2.2	61	-0.7

Scale of measurement: 1 = applicable till 6 = not applicable; *T* T-score, *df* degree of freedom, *SD* standard deviation; total n = 62-63 participants.

Further significant differences (using the 6 step scale) between the two subgroups were found, concerning “competence in grading”, “critical grading”, and “departmental improvements” (*see* Table 3).

**Table 3:** Differences between operative and non-operative disciplines in evaluation of examiner trainings, concerning further effects of the trainings

**Item**	**Professional discipline**	**N**	**Mean**	**SD**	**p-value**	**T**	**df**	**Mean difference**
The training improved my competence in grading	Operative	28	2.21	0.83				
	Non-operative	34	2.94	1.48	0.02	-2.44	54	-0.73
By the training my grading has become more critical	Operative	28	2.43	1.23				
	Non-operative	33	3.39	1.46	0.01	-2.81	59	-0.97
The training contributed to improvements in my departement (referring to oral examinations)	Operative	26	2.19	0.80				
	Non-operative	33	3.00	1.46	0.01	-2.71	52	-0.81

Scale of measurement: 1 = applicable till 6 = not applicable; *T* T-score, *df* degree of freedom, *SD* standard deviation; total n = 59-62 participants.

Overall, in six categories concerning oral examinations significant differences between participants from operative and non-operative disciplines were identified. These categories included “strengths and weaknesses of oral examinations”, “reliability”, “validity”, “competence in grading”, “critical grading”, and “departmental improvements”. In these issues, the impact of the training program was rated significantly higher by colleagues from operative specialties.

## Discussion

In reality, high quality oral examinations of medical students with precise pre-definition of relevant patient-oriented tasks and of the respective expectation levels cannot be taken for granted and are difficult to develop and to implement. Therefore, a structured training program for oral examiners in respect to the final medical exam has been implemented at the University of Ulm.

According to the present survey the trained examiners had the impression that passing of incompetent candidates is infrequent, but nevertheless an existing phenomenon during the oral part of the final exam. As the number of failing candidates in the oral exam is rather low, this may speak in favour of the candidates’ competence in general and consequently in favour of the quality of present curriculum. These results correlate well with the results of the written part of the final exam, centrally designed for the whole country, where the number of failing candidates is also rather low. For example, in fall 2011 182 students of the Medical Faculty of the University of Ulm participated in the written part of the final exam, with only three candidates failing. In the oral part none of them failed. A study performed by Seyfarth et al. (2010) compared the grades on the oral and written components of the final medical exam and proposed an improved concordance between the two components since 2002, when the actual German national medical licensing regulations came into force [10].

The dark side is represented by the fact that more than half of the examiners participating in our study nevertheless already had the experience of seeing incompetent candidates be passed on their oral exam. Passing incompetent candidates might endanger patient care and health. Consequently, the participating colleagues request to examine the candidates in the final exam more critically, by means of concrete clinical examples and clearly defined grading criteria. This is consistent with findings from the 1990′s that it seems to be far more difficult to rate bad or borderline performances during oral examinations than to rate good performances [11].

Surprisingly, the answers of the study participants belonging to operative disciplines turned out to differ significantly from the answers of the participants from non-operative disciplines. One rather provocative explanation for these differences could be that so far the colleagues from operative fields have not been familiar enough with the didactic theories concerning oral examinations and that subsequently higher learning and training effects could be achieved. On the other hand, for the colleagues from operative disciplines the trainings did not only lead to individual learning and training effects, but also to examination-related improvements in their respective departments. This might indicate that the surgical participants handled the newly acquired competences in a very active way.

A certain limitation of the study is the relatively low number of participants: more than 300 persons were addressed to participate in present survey, but only 63 persons answered the questionnaire – although factors reported to enhance the response rate were specifically addressed in the study, such as survey length (the questionnaire focused on only 28 items), design issues (clear layout), and research affiliation (cover letter by the Dean of Education of the Medical Faculty). This is partly explainable by the known high turnover of staff at University hospitals and by the well-known time restrictions of the target group, as recently outlined in this journal [12], and partly by the relative high frequency of such electronic surveys, leading to a certain “survey-fatigue” of the potential participants; response rates to online surveys have significantly decreased since 1986 as an effect of the population being “oversurveyed” [13,14].

The fact that workshop participation was voluntary might also have slightly biased the study results. When the training was initiated in 2007, the Medical Faculty voted for a bonus system instead of an obligatory participation; the bonus system offers a small financial incentive not to the attendees but to the respective departments. The word-of-mouth recommendation and the rather positive feedback of former participants result in consistent high participation numbers (also on the base of high staff turnover at the University Hospital). Meanwhile, almost all examiner novices participate in the training, either by intrinsic motivation or sent by the chairmen of their department.

Another limitation of the study results from the fact that answers to the survey items are based on self-assessment of the participants. The quality of self-assessment with its tendency towards under- or overestimation of competence has been discussed very differently throughout the literature, but trainings with expert feedback (as performed in present examiner trainings) have been reported to have the capacity to generate a good relation of self-assessment and objective reality [15-17].

Furthermore, the use of untrained examiners as an “objective” control group in the high-stakes situation of the final exam could not be considered a reasonable and acceptable alternative.

## Conclusion

The results of present study suggest that oral examinations in medicine may contribute substantially to patient safety, especially if three conditions are fulfilled:

1. The examinations are designed to be based on concrete clinical case examples

2. The grading is grounded on clear and objective criteria

3. The candidates are examined more critically than in the past

In order to prevent incompetent candidates from passing the oral examination, we have to continue to develop our examiner trainings, putting a main emphasis on these three conditions.

Since the training effects on the personal as well as on the institutional level are especially high for colleagues from the operative disciplines, these training programs need to be offered especially in surgical departments.

## Competing interests

The authors declare that they have no competing interests.

## Authors’ contributions

WO designed the questionnaire and wrote the study, SG performed the data handling and data analysis, MHL designed and supervised the study and helped writing the manuscript. All authors read and approved the final manuscript.
